# Arthroscopic debridement of the ankle for mild to moderate osteoarthritis: a midterm follow-up study in former professional soccer players

**DOI:** 10.1186/s13018-016-0368-z

**Published:** 2016-03-30

**Authors:** Leonardo Osti, Angelo Del Buono, Nicola Maffulli

**Affiliations:** Unit of Arthroscopy and Sports Trauma Surgery, Hesperia Hospital, Via Arquà 80/b, Modena, Italy; Department of Orthopaedics and Traumatology, Hospital Vaio, Via Tincati, Fidenza, Parma Italy; Department of Musculoskeletal Disorders, Faculty of Medicine and Surgery, University of Salerno, Salerno, Italy; Centre for Sports and Exercise Medicine, Barts and The London School of Medicine and Dentistry, Mile End Hospital, 275 Bancroft Road, London, E1 4DG UK

## Abstract

**Background:**

The aim of this study is to report the clinical and functional outcomes following arthroscopic management of anterior impingement, grade III–IV cartilage lesions, and mild to moderate osteoarthritis of the ankle in former soccer players.

**Methods:**

The study included 15 former male professional soccer players with mild to moderate degenerative changes of the ankle who had undergone arthroscopic debridement and management of secondary injuries of the ankle. Preoperatively and at the last follow-up, at an average of 7.4 years, the American Orthopaedic Foot and Ankle Society (AOFAS) and the Kaikkonen scales and visual analogue scale (VAS) assessment were administered to all patients. Ankle osteoarthritis was assessed from weightbearing anteroposterior and lateral radiographs of both ankles.

**Results and discussion:**

At the last follow-up, the average AOFAS score had increased significantly from 48 (range, 29–69) to 86 (range, 63–94) (*P* < 0.0001), with good to excellent scores in 11 patients (74 %). The average Kaikkonen preoperative score of 43 (range, 28–70) had significantly improved to 85 (range, 61–95) (*P* < 0.0001), with good excellent scores in 11 patients (74 %). VAS values were also improved at the last follow-up. At the last appointment, only one (7 %) patient had abandoned altogether any sport, as he did not feel safe with his ankle and he felt too old to continue.

**Conclusions:**

Anterior ankle arthroscopy for management of mild to moderate ankle arthritis is safe, effective, and low cost and allows former athletes to safely return to ordinary daily activities and recreational sport activities.

## Background

Joint space narrowing, osteophytes, cartilage lesions, and loose bodies are all features of ankle arthritis. This condition is post-traumatic in 70–80 % of instances, especially in athletes [1]. In soccer players, repetitive stresses and twisting injuries, even without frank traumatic insults, may cause it [2, 3], leading to “footballer ankle”, with anterior and posterior bone spurs, fibrous tissue impingement, and ligamentous instability [4].

At present, there is no consensus on the best management of this condition. Supramalleolar osteotomy, distraction arthroplasty, ankle arthrodesis, or total ankle replacement have all been performed successfully in patients with end-stage osteoarthritis [5]. In the early stages, in young patients, ankle arthroscopy may well improve symptoms and function, and prevent or delay major surgery [6]. Even though arthroscopy may be indicated for the management of early arthritis, there is little information about its effectiveness in patients with frank osteoarthritis [7, 8]. This study reports the clinical and functional outcomes of former soccer players who had undergone arthroscopic management for anterior impingement, grade III–IV cartilage lesions, and mild to moderate osteoarthritis of the ankle.

## Methods

This is a retrospective study including 15 male patients with mild to moderate degenerative changes of the ankle who had undergone arthroscopy from 2001 to 2010 at the Department of Arthroscopy and Minimally invasive surgery, Hesperia Hospital, Modena, Italy. The investigation was performed after approval of the Ethics Committee of the Hesperia Hospital, Modena, Italy. Patients were selected according to the following criteria: arthritis of the ankle with osteophytes and narrowing with no disappearance of the joint space (grade I–II degenerative changes according to the classification by Krips et al. [9]), major anterior impingement, full sized cartilage lesion, loose bodies, limited motion, inability to practice loading sports, pain and discomfort in daily activities, and unresponsive to conservative treatment. Exclusion criteria included complete obliteration of the ankle joint space, reflex sympathetic dystrophy (RSD) or extensive area of bone marrow edema, posterior impingement requiring posterior arthroscopy of the ankle, body mass index (BMI) over 30, cavus/varus foot deformity, and history of ankle fracture.

Of 108 patients who underwent ankle arthroscopy in the period 2001–2010 at our institution, 15 patients met the inclusion criteria, and were enrolled in the study: they had all been professional soccer players who had competed at least at county level, and whose main source of income had derived from soccer. The first author (LO) made the diagnosis in all patients by history, clinical examination, MRI findings, and confirmed it at arthroscopy in all instances. The anterior drawer test was evaluated bilaterally clinically, to exclude hypermobility. With the patients supine and the ankle in neutral position, a side-to-side difference in tibio-talar translation less than 5 mm was considered normal (grade 0), a side-to-side difference 5 to 10 mm was classified as grade I, a side-to-side difference 10 to 15 mm was classified as grade II, and a side-to-side difference greater than 15 mm was classified as grade III.

We considered the anterior drawer test as normal when a hard stop could be appreciated, and pathological when the stop was absent or soft. At anterior drawer testing, eight patients were normal, and seven presented grade 1 laxity. All patients underwent plain anteroposterior and lateral radiographs of the ankle and foot and, for objective evaluation, the American Orthopaedic Foot and Ankle Society (AOFAS) ankle-hindfoot scale [10], the Kaikkonen et al. ankle scoring scale [11], and the visual analogue scale (VAS) assessment were administered to each patient.

At the final assessment, a clinical research fellow (ADB) who had not been involved in the original management of the patients performed all the examinations, administered all the tests, and examined and classified all the images.

### Surgery

**Fig. 1:**
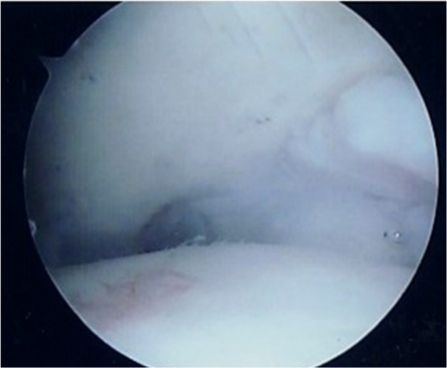
Arthroscopic view of grade IV cartilage lesion of the talus

The first author (LO) performed all surgical procedures, under general anesthesia, in the supine position, with manual or temporary body traction of the ankle, without a tourniquet. A standard knee arthroscope and two standard anteromedial and anterolateral portals were used, switching to a 2.7 mm 30° angled arthroscope if needed. At arthroscopy, cartilage lesions were classified according to Outerbridge (Fig. 1). All the structures were palpated with a probe to assess osteochondral and bony features, and the presence of soft tissue impingement.

**Fig. 2:**
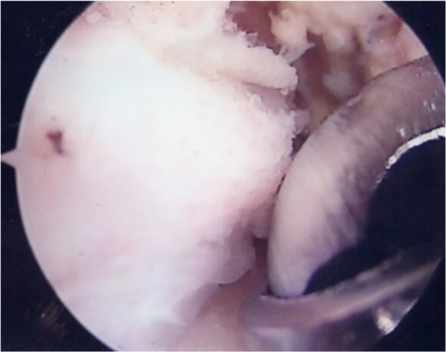
Arthroscopic image showing debridement of bony impingement

**Fig. 3:**
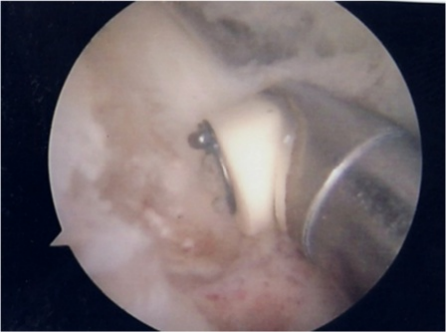
Arthroscopic image showing debridement of scar tissue with the radiofrequency probe

**Fig. 4:**
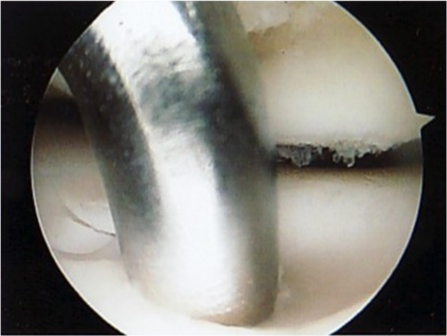
Arthroscopic image showing the position of the awl when performing microfractures. The tip of the awl is perpendicular to the bed of the lesion

**Fig. 5:**
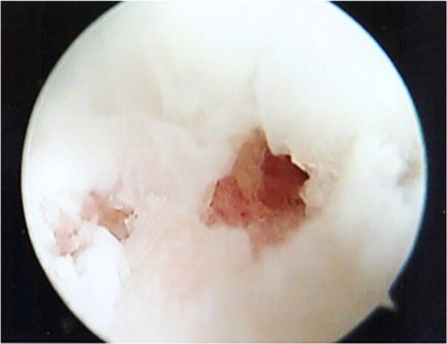
Image of the hole within the bone after microfracture

Appropriate bony (Fig. 2) and soft tissue debridement (Fig. 3) was performed to address anterior impingement in all patients using both a mechanical shaver and a radiofrequency probe. Loose bodies were removed in the seven patients; microfractures were undertaken in all instances for management of grade III–IV cartilage lesions. Specifically, after preparation of the cartilage bed, the lesion was measured using a scaled probe. An awl (Condropick, Arthrex, Naples, FL, USA) was introduced with the tip perpendicular to the bed of the lesion (Fig. 4), and the holes were made as described by Steadman, 3 to 4 mm apart and about 2 to 4 mm deep, starting from the center of the lesion to proceed peripherically. The residual stability of the cartilage was assessed, and the irrigation was temporarily stopped to ascertain that marrow fat droplets and blood came out from the holes (Fig. 5).

After surgery, all patients followed a homogeneous rehabilitation program. Specifically, active and passive motion exercises were started the day after surgery; partial weightbearing with crutches was prescribed for 6 weeks. Assisted physiotherapy was started after 2 weeks, avoiding exercises which induced pain. Balance board weightbearing exercises were allowed after 2 months, and loading activities were allowed after 3 months. Patients were allowed to return to sport after 6 months. Viscosupplementation of the ankle using medium weight hyaluronic acid was performed in all patients 2 to 4 months after surgery, based on the inflammatory status (synovitis) of the ankle joint. Patients were assessed thereafter at 6 month intervals for 2 years, and discharged at that stage.

### Follow-up

At the last examination, a mean of 7.4 years after surgery (range, 4–13 years; standard deviation, 2.31 years), all patients underwent conventional anteroposterior and lateral radiographs. Immediately before the operation and at the latest follow-up, all patients completed the AOFAS score, which was classified as excellent (90–100), good (80–89), fair (70–79), or poor (<70). We considered failure patients with an AOFAS score of <80. The Kaikkonen scoring system was also used, and classified as excellent (85–100), good (70–80), fair (55–65), or poor (<50). A patient with a Kaikkonen score of <70 was considered as failure. The VAS assessment was also administered to all patients to assess ankle pain; patients were also asked about their activity level.

Ankle osteoarthritis (OA) was assessed from weightbearing anteroposterior and lateral radiographs of both ankles, obtained preoperatively and at the final follow-up. The system described by Krips et al. [9] was used, which classifies the joint as grade 0 (normal), grade I (in the presence of osteophytes without joint space narrowing), grade II (when the joint space is narrowed), and grade III (if the space is markedly reduced or deformed). Radiographs were assessed by the clinical research fellow. In more demanding cases, the diagnosis was confirmed by a radiologist not involved in the study, and experienced in muscle, tendon, and ligament disorders in athletes.

### Statistics

After the assessment of normality with the Kolmogorov-Smirnov test, the Wilcoxon signed-rank test was used to compare preoperative and postoperative AOFAS, Kaikkonen, and VAS values. A Pearson’s chi-square test was used to test the association between the status of postoperative sport activity and the grade of ankle OA. A *P* value of <0.05 was considered to be statistically significant. All analyses were performed using SPSS software, version 13.0 (SPSS, Chicago, Illinois).

## Results

All 15 patients included in our original cohort returned to our clinic at the average final follow-up of 7.4 years. The average age at surgery was 42 (from 38 to 45; SD 2.3; median 44); the mean time of activity before the onset of symptoms had been 27 years (from 24 to 33; SD 2.7; median 26). Patients were operated on after an average of 8 months (from 6 to 12 months; SD 1.9; median 7) after the onset of symptoms. The right ankle was involved in 11 (67 %) patients, and the left in four (33 %) patients. The AOFAS significantly improved from an average preoperative score of 48 (range, 29–69; SD 11.6; median 48) to 86 (range, 63–94; SD 7.4; median 88) at the last follow-up (*P* < 0.0001). Postoperatively, the AOFAS scores were graded as excellent in seven (47 %), good in four (27 %), fair in three (20 %), and poor in one (6 %) patients. The Kaikkonen score significantly improved from an average preoperative score of 43 (range, 28–70; SD 12.5; median 40) to 85 (range, 61–95; SD 8.2; median 88) at the last follow-up (*P* < 0.0001). Postoperatively, the Kaikkonen score was graded as excellent in eight (53 %), good in three (20 %), fair in three (20 %), and poor in one (6 %) patient. VAS values also improved from a baseline average value of 7.1 (range, 5–10; SD 1.5; median 7) to an average of 2.9 (range, 0–6; SD 1.6; median 3) at the last follow-up.

At the last appointment, eight (53 %) patients practiced recreational high-impact sport activities (soccer, tennis, and running) an average of two times per week; six (40 %) practiced recreational non-weightbearing sport activities (cycling and swimming) an average of two times per week; and 1 (7 %) abandoned altogether sport, as he did not feel safe with his ankle and he felt too old to continue. No surgical complication was encountered.

### Imaging outcomes

At the time of surgery, the ankle of all patients demonstrated radiographic signs of degenerative changes: eight patients exhibited grade I changes, and seven patients exhibited grade II changes. At the final follow-up, seven patients exhibited radiographic signs of grade I degenerative changes of the ankle, and five had radiographic signs of grade II degenerative changes of the ankle, three had evolved to grade III osteoarthritis. One of these three patients (33 %) had abandoned sport activity, and two (67 %) practiced recreational low-impact sports such as swimming and cycling.

## Discussion

The main finding of the present study is that ankle arthroscopy and concomitant arthroscopic management of secondary injuries, when performed in selected patients with mild to moderate degenerative changes to the ankle, provide high rates of satisfaction and good functional results with positive impact on the quality of life.

There is strong evidence in support of ankle arthroscopy in patients with arthrofibrosis, synovitis, arthritis, fractures, and osteochondral defects [12]. Specifically, in osteochondral lesions, arthroscopic debridement and microfractures provide very satisfying results in the midterm, in terms of AOFAS and VAS values [13]. In patients with anterior bony impingement, at a mean follow-up of 9 years, the AOFAS scores were still significantly improved, but age at surgery, radiographic changes, and concomitant cartilage lesions were negative prognostic factors [14].

We performed microfractures in all patients for management of grade III–IV cartilage injuries. In appropriately selected cases, microfractures produce stable regenerated fibrocartilage, reduce pain and crepitus, and improve the gliding of the articular surfaces. They are easy to perform, safe, effective in the short- and midterm, but, in the long term, outcomes decline [15]. Nevertheless, at a minimum follow-up of 8 years after arthroscopic microfractures, good-to-excellent results were observed in 78 % of patients, with no poor results, and low progression of osteoarthritis (33 % grade I progression) [16].

A recent systematic review reported that, in patients with osteochondral lesions of the talus, arthroscopic debridement and microfractures provides 80 % of good to excellent results on average [17].

When evaluating prognostic factors, arthroscopy may be effective if cartilage lesions are small, and involve the lateral side of the talus [18].

In our study, all patients presented degenerative changes of the ankle. Ankle arthroscopy, though effective for isolated osteochondral lesions, provides less encouraging results in patients with frank osteoarthritis [8, 19]. In the knee, arthroscopic debridement has no value in the management of arthritis [20]. In the ankle, the pattern and evolution of cartilage lesions is different compared to the knee [21], given the different microscopic and biomechanical properties, content of proteoglycan and water, and altered response to catabolic factors [22]. Therefore, selected groups of patients may benefit after ankle arthroscopy, in terms of pain relief and functional improvement [6, 23]. More complex and invasive procedures such as supramalleolar osteotomy, ankle arthrodesis, or joint arthroplasty may be delayed or avoided [24].

In advanced osteoarthritis, in the midterm, microfractures do not affect the outcomes [25]. Nevertheless, they decrease the risk of clinical failure, and improve the outcomes compared to debridement alone [26]. Therefore, it is essential to address associated intra-articular lesions, present in up to 72 % of patients [27, 28], and predictive of poor results.

There is no consensus on postoperative rehabilitation and time to return to activity after ankle arthroscopy [29]. We point out that all our patients returned to full weightbearing 6–8 weeks after the index surgery, to allow osteoblasts to form new woven bone and chondroblasts to produce a matrix containing type II collagen and proteoglycans, all forming fibrocartilaginous tissue. After this time, a hyaline-like cartilage with a high component of type II collagen can be detected [30], and the osteochondral defects are completely filled with mostly hyaline-like tissue [31].

All our patients underwent viscosupplementation with hyaluronic acid. The most recent evidence on this matter states that viscosupplementation does not influence the natural progression of osteoarthritis, but it reduces pain and improves function [32].

There are limitations to this study. First, it was a retrospective and nonrandomized investigation. Although the retrospective design of the study and the absence of a control group do not allow us to draw definitive conclusions, this is the first study to report on midterm outcomes in former professional soccer players who had undergone arthroscopic procedures for management of mild to moderate osteoarthritis of the ankle. We are aware that the AOFAS scale is not validated, and that other scales are available to assess ankle function [33], but it was a common practice to administer it at the stage the study had been conceived, and we have successfully used it for the assessment of patients with ankle disorders [27, 28]. The strength of the study was that we enrolled a selected group of patients according to strict selection criteria, excluding all confounding factors of bias. Although the analysis was retrospective, data collection was longitudinal. The first author, a fellowship-trained surgeon, performed all surgical procedures, the rehabilitation protocol was homogeneous, an independent investigator not involved at the index surgery examined the patients at the last follow-up, and none of the patients was lost to the follow-up.

## Conclusions

**Fig. 6:**
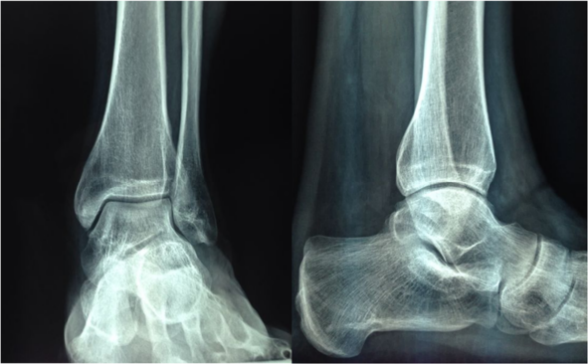
Anteroposterior and lateral radiographs of the left ankle in a soccer player showing signs of moderate osteoarthritis of the ankle

**Fig. 7:**
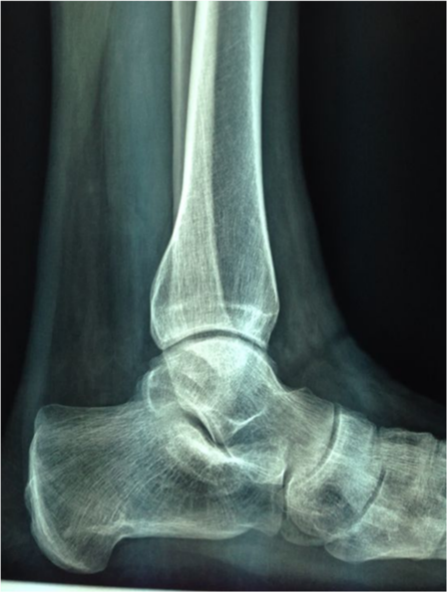
Lateral radiograph of the left ankle of the same patient at 7-year follow-up, showing no progression of degenerative changes

In former professional soccer players with mild to moderate ankle arthritis, microfractures, and concomitant arthroscopic management of associated injuries are safe, effective, and low cost, allowing them to return safely to ordinary daily and recreational sport activities. In this series, good excellent outcomes were observed in more than 70 % of patients, and radiographic degenerative changes progressed in two patients (13 %) (Figs. 6 and 7). Appropriately powered studies with longer follow-up, possibly testing different treatment modalities and different rehabilitation protocols are nevertheless needed.
